# Effect of Oak Powdery Mildew on Ascorbate–Glutathione Cycle and Other Antioxidants in Plant—*Erysiphe alphitoides* Interaction

**DOI:** 10.3390/cells13121035

**Published:** 2024-06-14

**Authors:** Monika Skwarek-Fadecka, Justyna Nawrocka, Katarzyna Sieczyńska, Jacek Patykowski, Małgorzata Maria Posmyk

**Affiliations:** 1 Department of Plant Ecophysiology, Faculty of Biology and Environmental Protection, University of Lodz, Banacha 12/16, 90-237 Łódź, Poland; malgorzata.posmyk@biol.uni.lodz.pl; 2 Department of Plant Physiology and Biochemistry, Faculty of Biology and Environmental Protection, University of Lodz, Banacha 12/16, 90-237 Łódź, Poland; justyna.nawrocka@biol.uni.lodz.pl; 3 Łukasiewicz–Łódź Institute of Technology, Zgierska 73, 91-463 Łódź, Poland; katarzyna.sieczynska@lit.lukasiewicz.gov.pl; 4 Independent Researcher, 90-008 Lodz, Poland; jacpat908@gmail.com

**Keywords:** oak powdery mildew, ascorbate–glutathione cycle, phenolic compounds, lignin, metals

## Abstract

*Erysiphe alphitoides* is a species of powdery mildew responsible for the major foliar disease of oak trees, including *Quercus robur*. Infection with *E. alphitoides* leads to a reduction in the growth of the trees and in their ability to survive. This paper reports on the biochemical changes characteristic of defence responses in oak leaves with different infection area sizes, collected in July, August, and September during three growing seasons. The study highlights the effect of *E. alphitoides* infection on changes in the ascorbate-glutathione cycle, phenolic compound profile, and metal content (mineral distribution). Visible symptoms of pathogen infection appeared gradually in July, but the most intense biochemical plant responses in oak leaves were detected mainly in August and September. These responses included increased ascorbate-glutathione enzyme activities, phenolic compounds, and metal contents. In addition, microscopic analyses revealed a strong fluorescence signal of lignin in the epidermis of pathogen-infected leaves. The involvement of the studied compounds in the basic defence mechanisms of oak against *E. alphitoides* infection is discussed in the paper.

## 1. Introduction

The pedunculate oak (*Quercus robur* L.) is one of the most valuable deciduous tree species in Poland and the most susceptible to infection with the pathogen *Erysiphe alphitoides* [(Griffon and Maubl.) U. Braun and S. Takam.], which causes oak powdery mildew, one of the most important foliar diseases in Europe [1]. *E. alphitoides* is a non-indigenous fungal species from North America that was first reported in Europe (France) in 1907 [2]. The first occurrence of *E. alphitoides* was documented in Poland in 1909 [3,4]. Within a few years, the disease had spread throughout Europe. Erysiphales are obligate biotrophic fungi with a wide range of common species to approximately 10,000 plant species. Other Erysiphaceae species found on oak leaves include *E. abbreviata* (Peck) U. Braun and S. Takam., *E. calocladophora* (G.F. Atk.) U. Braun and S. Takam, *E. extensa* (Cooke and Peck) U. Braun and S. Takam., *E. gracilis* R.Y. Zheng and G.Q. Chen, *E. hypophylla* (Nevod.) U. Braun and Cunningt, *E. quercicola* S. Takam. and U. Braun, and *E. polygoni* D.C. [4]. Moreover, the strong increase in *E. alphitoides* abundance suggests that *E. alphitoides* has caused the partial displacement of *E. quercicola*. *E. alphitoides* may have been introduced a few years after *E. quercicola*, which grew exponentially and spread [5]. In Poland, according to Sucharzewska (2009) [6] and Roszak et al. (2019) [7], there are *E. alphitoides* and *E. hypophylla* (more common in the western part of the country). The two parasites can either occur separately or together on the same plant and even on the same leaf. The upper surface of the leaf is usually colonised by *E. alphitoides* and the lower surface by *E. hypophylla*. Depending on the species of oak, powdery mildew was reported to have different effects [8,9]. The pathogen *E. alphitoides* mainly infects *Q. robur* L., *Q. petraea* (Matt.) Liebel., *Q. conferta* (Kit.) Vuk., *Q. pubescens* Willd., *Q. cerris* L. and *Q. pyrenaica* Willd. [1,7]. In addition, the pathogen has also been identified on the leaves of tree species such as *Fagus sylvatica* L., *Castanea sativa* Mill., or *Eucalyptus gunnii* Hook. f. [7]. The epidemic of powdery mildew in Europe usually starts in late spring, when the shoots are developing [10]. Changes induced by this type of biotrophic pathogen include a reduction in net carbon assimilation and leaf longevity, which is associated with reduced radial growth or even increased mortality, especially in young seedlings [11].

Plant infections and biotic stress are caused by a number of pests, bacteria, viruses, and fungi. Fungal pathogens are one of the most problematic agents causing biotic stress to plants, especially under conditions of increased humidity, which allows their proliferation and spread in the environment. To overcome pathogens, plants have developed various defence mechanisms and many physio-biochemical strategies to protect themselves against their attack [12]. One of the defence mechanisms of plants, including trees, against disease is the accumulation of phenolic compounds such as lignin, which increases during plant-pathogen interactions [13,14]. Plants accumulate cell wall aposites at sites of invasion by biotrophic fungi. Lignin, as one of the major components of the cell wall of vascular plants, is the first line of defence against the successive penetration of invasive pathogens. Lignification is essential for the structural integrity of the plant cell wall and is crucial for plant development as well as a process that occurs in response to stress. The biotic stresses can lead to the formation of different reactive oxygen and nitrogen species (ROS and RNS) in plant cells, which, at low concentrations, can generate a stress-specific signal that modulates the plant’s response to a particular stress or combination of stresses [15,16]. A common response of plants, including trees, to the maintenance of balanced ROS levels under stress, is the activation of the enzymatic and non-enzymatic antioxidant systems [17]. In addition, the plant hypersensitive response (HR) is a rapid, localised cell death that occurs at the site of pathogen invasion and is associated with disease resistance. ROS may promote the biosynthesis and accumulation of salicylic acid (SA), which is involved in the induction of systemic acquired resistance (SAR) in plants. SA plays an essential role in the signalling activated by plant pathogen-associated molecular patterns (PAMPs) [14,18,19]. Stress-activated increases in the content of phytohormones such as jasmonic acid (JA), ethylene (ET), and SA involved in various signalling pathways are pathogen-induced responses [14,20,21]. Regarding *E. alphitoides* influence on *Q. robur*, it has been established that the pathogen may (i) disrupt chlorophyll and cell walls by degradation of cellulose and pectin, (ii) decrease physiological and antioxidative parameters and water use efficiency, (iii) increase secondary metabolites and polyamine content, (iv) as well as lipid peroxidation [22,23]. In our previous study [24] on the interaction between *Q. robur* and *E. alphitoides*, we showed that H_2_O_2_ and NO, which are considered to be important signalling molecules of plant defence and resistance, were accumulated to different extents in infected leaves. Furthermore, lipoxygenase (LOX) products, isoprenoids, and methyl salicylate were the most abundant volatile organic compounds (VOCs) produced by all infected leaves. The antioxidant defence system that prevents secondary oxidative stress includes many enzymatic components, such as superoxide dismutase (SOD), catalase (CAT), peroxidases (POX), ascorbate peroxidase (APX), monodehydroascorbate reductase (MDHAR), dehydroascorbate reductase (DHAR), and glutathione reductase (GR), and non-enzymatic low-molecular-weight components such as ascorbic acid (AsA), glutathione (GSH), phenolic compounds, lignin, carotenoids, free amino acids, and α-tocopherols [25,26]. The enzymatic antioxidants are mainly involved in either preventing the Haber-Weiss reaction or the Foyer-Halliwell-Asada pathway, which reduces H_2_O_2_ and utilises the reduction potential of NADPH [26]. On the other hand, non-enzymatic antioxidants not only protect various cellular elements from oxidative damage but also play an important role in plant development by stimulating cell division (mitosis) and growth [27,28].

Considering all the above, the objectives of the present study were as follows: (1) to determine the changes in the activities of enzymes of the ascorbate-glutathione cycle, including APX, DHAR, MDHAR, and GR; (2) to determine the contents of ascorbate, glutathione, and phenolic compounds; and (3) to determine the contents of different metals in *Q. robur* leaves. All the presented parameters are considered important elements and markers of defence responses, the analysis of which may provide new information on the biochemical basis of plant protection against *E. alphitoides*.

## 2. Materials and Methods

### 2.1. Collecting Materials and Designing Experiments

One-year-old oak (*Quercus robur*) seedlings obtained from the Sowin Forestry container nursery (Gidle Forest District, Poland, 50°58′41.4″ N; 19°28′36.8″ E) were used for the experiment. Plants were collected every 28 days (±2 days) after the first natural appearance of infection symptoms on the leaves (white, powdery spots) over the course of three growing seasons, in July, August, and September (hereafter referred to as three-time points). The influence of the environment during the experiment was limited to temperature changes during the day and night. Average monthly temperatures varied around 19–20 °C (July), 20 °C (August), and 15 °C (September) (data from the National Hydrological and Meteorological Service). The soil substrate was crushed with highly acidic peat supplemented with perlite and other minerals (Table 1).

Powdery mildew fungus was identified as *E. alphitoides* based on morphological features that were described previously by Skwarek-Fadecka et al. [24]. Microscopic analysis of leaves showing signs of fungal infection (a white powdery coating on the upper and/or lower leaf surface) revealed the presence of mycelium, conidiophores, and conidia associated with powdery mildew (Erysiphales). The pathogen was identified as *Erysiphe alphitoides* (Griffon and Maubl.; U. Braun and S. Takam.) [29]. It differed from two other oak parasites found in Europe: *E. quercicola* (S. Takam. & U. Braun) and *E. hypophylla* (Nevod.; U. Braun & Cunningt.) in ascomatal and conidial characters, respectively [30]. To assess the extent of fungal infection on the leaf surface (i.e., the percentage of leaf area covered by white fungal hyphae of *E. alphitoides*), as previously described by Copolovici et al. (2014) [31], the leaf was photographed and the infected area analysed using image analysis software, Motic Images Plus 2.0ML (Motic China Group, Hong Kong, China, Asia), according to the manufacturer’s instructions. 

The experiment was carried out on young leaves at the top of the seedling. Leaves from four groups of plants were examined: (I) seedlings with no visible symptoms of disease (control plants, C), (II) seedlings infected with *E. alphitoides*, with infection covering less than 5% of the leaf area (<5% Inf), (III) seedlings infected with *E. alphitoides*, with infection covering 12–15% of the leaf area (12–15% Inf), and (IV) seedlings infected with *E. alphitoides*, with infection covering 25% of the leaf area (25% Inf).

For all biochemical analyses, whole leaves from each variant (groups I–IV) were frozen in liquid nitrogen before the appropriate amount of leaf tissue was transferred to the subsequent sample preparation steps.

### 2.2. Leaves Moisture Content Measurements

Fresh weight (FW) and dry weight (DW) were measured on leaves of the same size. FW was measured immediately after harvest. The DW was determined by drying the plant material in paper envelopes at 60 °C for at least 48 h. From the FW, the moisture content (MC) was calculated according to Ellis et al. [32] using the following formula:

MC [%] = [(FW − DW)/FW] × 100(1)

### 2.3. Metal Content Determination

The metal content was measured using the method previously described by Skwarek et al. [33]. Whole leaf tissues (0.2–0.5 g) were placed in a Teflon vessel, and 6 mL of HNO_3_ (65%, *w*/*w*, Chempur, Piekary Śląskie, Poland) was added. The leaves were mineralised using a Magnum II microwave mineraliser (Ertec, Wroclaw, Poland). The mineralisation process was carried out in three cycles for a total of 20 min at a maximum temperature of 300 °C and a maximum pressure increasing to 45 bar, with maximum microwave power (100%). The clear mineralizates were quantitatively transferred to 25 mL flasks. The flasks were made up of demineralised water. Multi-element analysis was performed by inductively coupled plasma optical emission spectrometry using an ICP-OES 5110 spectrometer (Agilent, Santa Clara, CA, USA). Commercial argon (from HenDuKol, Łódź, Poland) was used for plasma generation. After sample digestion, the ICP-OES quantification procedure was performed in the axial view of the plasma with a radiofrequency power of 1400 W, sample flow rate of 1.4 mL min^−1^, plasma gas flow rate of 12 L min^−1^, auxiliary gas flow rate of 1.0 L min^−1^, nebuliser gas flow rate of 0.7 L min^−1^, and integration time of 3 × 10 s. The determinations were made using the external standard method. The contents of the metals tested in the samples were read from the standard curves generated from the multi-element standard ICP solution (metals: Al, Ca, Cr, Cu, Fe, K, Mg, Mn, Na, Ni, P, and Zn in 5% HNO_3_, LGC Standard, Manchester, NH, USA) by appropriate dilution of the standards with 5% HNO_3_ (*v*/*v*). Calibration solutions used for ICP-OES determinations were prepared by serial dilution of multi-element stock solutions containing 1000 mg L^−1^ of each analyte. The content of each element was expressed as mg kg^−1^ DW.

### 2.4. Determination of Ascorbate–Glutathione Cycle Enzymes Activities

To prepare plant extracts for the determination of enzyme activities, including APX, DHAR, MDHAR, and GR, 1 g of whole leaf tissue was homogenised in 10 mL of 100 mM phosphate buffer, pH 7.0. The extraction mixture contained 1 mM edetic acid (EDTA), 1 M NaCl, 1% polyvinylpolypyrrolidone (PVPP), and 1 mM sodium ascorbate. The homogenates were centrifuged at 4 °C (20,000× *g*/20 min) and the supernatants were used immediately for enzymatic analyses. 

Ascorbate peroxidase (APX, EC 1.11.1.11) activity was determined after oxidation of ascorbate at 265 nm (ε = 13.7 mM^−1^ cm^−1^) according to a modified method of Nakano and Asada [34]. The reaction mixture (2 mL) contained 50 mM potassium phosphate buffer (pH 7.0), from 0.02 to 0.05 mL of the enzyme extract, and 0.05 M of sodium ascorbate. The reaction was initiated by the addition of 0.05 mM of H_2_O_2_. APX activity was expressed as [μmol_DHA_ min^−1^ mg^−1^ _protein_]. 

Determination of dehydroascorbate reductase (DHAR, EC 1.8.5.1) activity was performed following the formation of ascorbate at 265 nm (ε = 14.6 mM^−1^ cm^−1^) according to a method of Dalton et al. [35]. The reaction mixture (2 mL) contained 50 mM potassium phosphate buffer (pH 6.4), 2 mM glutathione (GSH), 0.05 mL of the enzyme extract, and 1.6 mM dehydroascorbate (DHA). DHAR activity was presented as [μmol_AsA_ min^−1^ mg^−1^_protein_].

Monodehydroascorbate reductase (MDHAR, EC 1.6.5.4) activity was determined following the oxidation rate of NADPH at 340 nm (ε = 6.22 mM^−1^ cm^−1^) according to a modified method of Hossain et al. [36]. The reaction mixture (2 mL) contained 50 mM Tris-HCl buffer (pH 7.2), 0.2 mM NADH, 2.5 mM AsA, 0.3 units AsA oxidase (EC 1.10.3.3), and 0.05 mL of the enzyme extract. MDHAR activity was presented as [-nmol_NADPH_ min^−1^ mg^−1^ _protein_]. 

Glutathione reductase (GR, (EC 1.6.4.2) activity was assayed by the determination of GSSG-dependent oxidation of NADPH at 340 nm (ε = 6.22 mM^−1^ cm^−1^) as described by Schaedle and Bassham [37]. The reaction mixture (2 mL) contained 50 mM Tris-HCl buffer (pH 8.0), 1 mM EDTA, 3 mM MgCl_2_, distilled H_2_O, 0.5 M oxidised glutathione (GSSG), 0.15 mM NADPH, and 0.1 mL of the enzyme extract. GR activity was presented as [-nmol_NADPH_ min^−1^ mg^−1^ _protein_].

### 2.5. Protein Content Assay

Protein content for the enzymatic assay was determined by the Bradford method [38] using a standard curve (in the range 0–50 µg) prepared with bovine serum albumin (Sigma-Aldrich, St. Louis, MO, USA). Absorbance was measured at 595 nm, and values were expressed as [mg mL^−1^].

### 2.6. Ascorbate Pool Determination

Reduced ascorbate (AsA), dehydroascorbate (DHA), and total ascorbate (AsA + DHA) contents were determined by the bipyridyl colorimetric method of Okamura [39], as described by Knȍrzer et al. [40]. Here, 1 g of whole leaf tissue was homogenised in 10 mL of 10% trichloroacetic acid (TCA). The homogenates were centrifuged at 4 °C (15,000× *g*/15 min). The determination of AsA + DHA content was performed by adding dithiothreitol (DTT) to reduce DHA to AsA in the samples. The DHA content was calculated by subtracting the AsA value from the total ascorbate. The contents of AsA, DHA, and AsA + DHA were expressed as [μmol g^−1^_FW_] and evaluated using the standard curve prepared for AsA (Sigma-Aldrich, St. Louis, MO, USA).

### 2.7. Glutathione Pool Determination 

Reduced glutathione (GSH), glutathione disulfide (GSSG), and total glutathione (GSH + GSSG) contents were determined by the colorimetric 5,5′-dithiobis(2-nitrobenzoic acid)-related (DTNB) method modified by Brehe and Burch [41]. A whole leaf tissue (0.5 g) was homogenised in 10 mL of 1% sulfosalicylic acid. The homogenates were centrifuged at 4 °C (15,000× *g*/15 min). The GSSG content was determined by adding 2-vinylpyridine to remove GSH. The GSH content was calculated by subtracting the GSSG value from the total glutathione. The contents of GSH, GSSG, and the total glutathione were expressed as [μmol g^−1^_FW_] and evaluated using the standard curve prepared for GSH (Sigma-Aldrich, St. Louis, MO, USA).

### 2.8. Determination of Different Phenolic Compounds Content

For the determination of total phenolics (PCs), phenylpropanoids (PP), and flavonoids (FL) contents, 250 mg of whole leaf tissue was homogenised in 5 mL 80% MeOH and centrifuged at 4 °C (20,000× *g*/20 min). For the determination of anthocyanins (ATH), 250 mg of whole leaf tissue was homogenised in 5 mL 1% HCl in 80% MeOH and centrifuged at 4 °C (20,000× *g*/20 min). Then, 1 mL of 1% HCl in 80% MeOH was added to the pellets remaining in the centrifuge tubes. After centrifugation at 4 °C (20,000× *g*/20 min), the extraction was repeated.

The PC content was determined by the Folin-Ciocalteu method based on the reduction of a phosphowolframate-phosphomolybdate complex by phenolics to blue reaction products, according to Singleton and Rossi [42]. Absorbance was measured at 725 nm. PC contents were calculated using a standard curve prepared for chlorogenic acid (Sigma-Aldrich, St. Louis, MO, USA) and expressed as [mg g^−1^_FW_]. 

The PP content was measured in the crude extract obtained by mixing the supernatant with 0.1% HCl in 95% ethanol and 2% HCl, 1:18 (*v*:*v*), according to Glories [43]. Absorbance was measured at 320 nm. PP content was calculated from a caffeic acid standard curve (Sigma-Aldrich, St. Louis, MO, USA) and expressed as [mg g^−1^_FW_].

The FL content was estimated by the modified method of Chang et al. [44]. The reaction mixture containing methanolic extract, 10% AlCl_3_ × 6H_2_O, and 1 M CH_3_COONa was incubated for 30 min at room temperature. Absorbance was measured at 234 nm. FL content was expressed as mg of the quercetin standard (Sigma-Aldrich, St. Louis, MO, USA) g^−1^ _FW_.

The ATH content was measured according to the method of Hodges and Nozzolillo [45]. Absorbance was estimated at 535 nm. The ATH content was calculated from the molar absorption coefficient for cyanidin 3-glucose (ε = 33 mM^−1^ cm^−1^) and expressed as μg g^−1^ _FW_.

### 2.9. Determination of Lignin Content and Histochemical Detection

Lignin was extracted according to the method of Bruce and West [46] and Mandal [47], with some modifications. To prepare supernatants, 0.5 g of whole leaf tissue was homogenised in 80% MeOH and centrifuged at 4 °C (15,000× *g*/20 min). The pellet was washed three times with 80% MeOH and dried at 60 °C for 24 h. The insoluble residue (15 mg) was mixed with 5 mL of 2 M HCl and 0.5 mL of TGA, boiled in a water bath for 4 h, and centrifuged (10,000× *g*/20 min). The supernatant was drained out. The pellet was washed twice with distilled water, then suspended in 2.5 mL of 0.5 M NaOH, and shaken on an orbital shaker for 2 h at 25 °C followed by centrifugation (10,000× *g*/20 min). The supernatant was then mixed with 1 mL of concentrated HCl and thioglycolic acid (TGA) (90:1; *v*:*v*) and allowed to precipitate for 4 h at 4 °C. An orange-brown pellet obtained after discarding the supernatant was dissolved in 0.5 N NaOH. After measuring the absorbance at 280 nm, the quantification was performed using the calibration curve for the lignin standard (alkali, 2-hydroxy-propyl ether), and the content was expressed as μg g^−1^ _FW_.

Histochemical detection of lignin deposition was prepared according to Drnovšek et al. [48]. Briefly, lead tissue fragments were washed with 80% EtOH and stained with 1% acridine orange prepared with 96% EtOH for approximately 30 min. The samples were washed in 25% HCl and immediately observed using a confocal laser scanning microscope system (Leica TCS SP8; Leica Microsystems, Mannheim, Germany), with a UV filter and excitation and emission wavelengths of 488 and 540 nm, respectively. Slides were scanned using the Leica LAS-AF programme, version 3.3.0.

### 2.10. Statistical Analysis

Data from the 3 independent growing periods (material collected in 3 months of each year) are presented as the average of at least 4–6 physiological replicates in each leaf variant. Two-way ANOVA was used to estimate the effects of infection intensity and time point (month) on all the parameters studied. Mean values were compared using the Tukey post hoc test. Differences were considered significant at *p* < 0.05. Significant differences are indicated by lowercase letters. All statistical analyses were performed using Statistica 13.1 software (TIBCO Software, Palo Alto, Santa Clara, CA, USA).

## 3. Results

### 3.1. Effect of Oak Powdery Mildew on the Moisture Content of Oak Leaves

**Figure 1:**
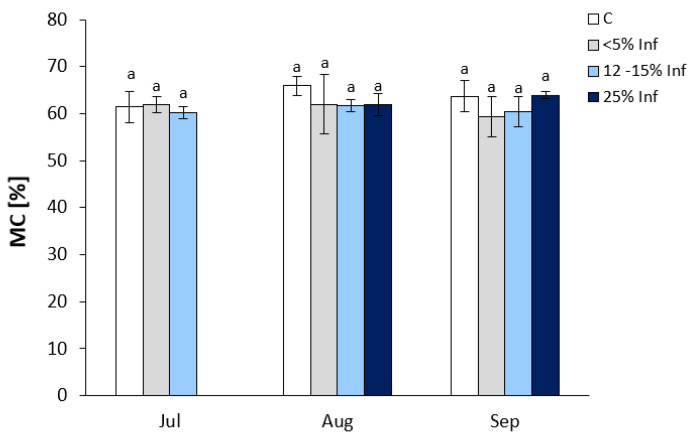
Moisture content of control and *E. alphitoides* infected oak leaves collected at fixed time points (July, August, and September) of three growing seasons. Values represent the means of 15–18 measurements per variant (C, <5% Inf, 12–15% Inf, 25% Inf) at each time point ±SD. Analysis of variance (ANOVA) for all parameters was followed by the Tukey post hoc test, and statistically significant differences (*p* ≤ 0.05) are marked using low letters.

No significant changes in MC were observed in the infected leaves compared to the respective controls (Figure 1). In July, oak leaves infected with *E. alphitoides* showed an increase in <5% Inf and a decrease in 12–15% Inf compared to controls. In leaves obtained in August, a decrease in MC was observed in the <5% Inf, 12–15% Inf, and 25% Inf categories compared to the control. In September, an increase in MC was observed only in 25% Inf.

### 3.2. Changes in Metal Contents

**Figure 2:**
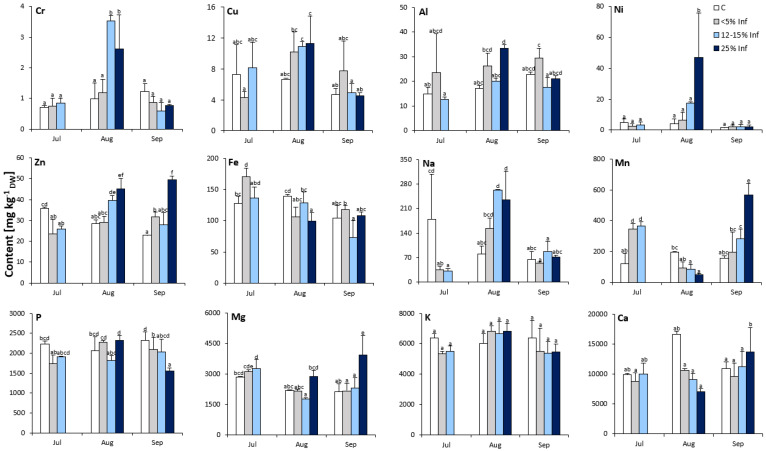
Elemental composition (Cr, Cu, Al, Ni, Zn, Fe, Na, Mn, P, Mg, K, Ca) of control and *E. alphitoides* infected oak leaves collected at fixed time points (July, August, and September) of three growing seasons. Values represent the mean of 9 analyses per variant (C, <5% Inf, 12–15% Inf, 25% Inf) at each time point ±SD. Analysis of variance (ANOVA) for all parameters was followed by the Tukey post hoc test and statistically significant differences (*p* ≤ 0.05) are marked using low letters.

The analysis revealed significant effects of the *E. alphitoides* infection on the elemental composition of the oak leaves (Figure 2). Interestingly, Cr, Ni, Zn, Mn, and Mg contents were significantly affected by the time point and degree of infection. Concerning the elemental content of oak leaves, only K was not affected by *E. alphitoides* infection at all time points. In July, the contents of Cr, Cu, Al, Ni, P, Mg, and Ca were more or less stable and were not significantly affected by *E. alphitoides* infection. Furthermore, at this time point, the results showed significantly increased contents of Mn in the <5% Inf and 12–15% Inf tested groups and of Fe in the <5% Inf tested groups, and significantly decreased contents of Zn and Na in infected leaves, compared to the respective controls. In August, the content of seven elements (Cr, Al, Ni, Zn, Na, Mn, and Fe) was significantly affected by *E. alphitoides* infection. The pathogen caused an increase in Cr, Zn, and Na in the 12–15% Inf and 25% Inf groups, and an increase in Al and Ni in the 25% Inf group. At the same time, *E. alphitoides* infection decreased Mn in 12–15% Inf and 25% Inf and Fe in 25% Inf. Other elements (Cu, P, Mg, and Ca) were not significantly affected by *E. alphitoides* infection. In September, the contents of Cr, Cu, Al, Ni, Fe, and Na were more or less stable and were not significantly affected by *E. alphitoides* infection. Compared to the respective controls, a significant increase in Zn was observed in the 5% and 25% Inf groups, as well as in Mn, Mg, and Ca in the 25% Inf group. At the same time, *E. alphitoides* infection decreased the P content in the 5% Inf and 25% Inf test groups compared to the respective controls.

### 3.3. Effect of E. alphitoides Infection on the Activities of Ascorbate-Glutathione Cycle Enzymes

The activities of all ascorbate-glutathione cycle enzymes in oak leaves showed significant infection-induced changes. The intensity of the changes for each enzyme was dependent on the degree of infection and time points.

**Figure 3:**
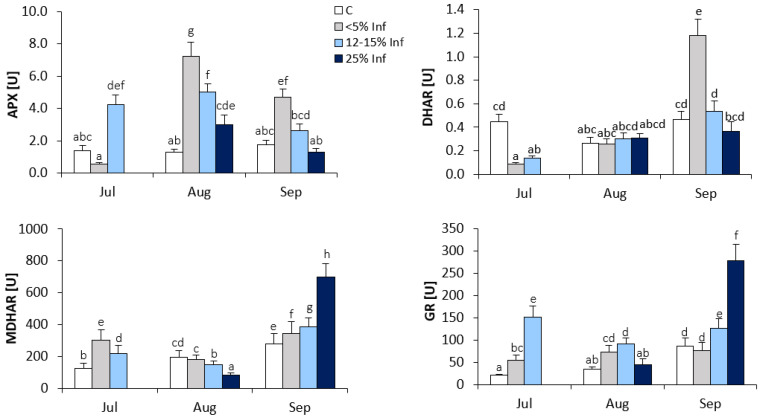
Changes in the activity of ascorbate-glutathione cycle enzymes (APX, DHAR, MDHAR and GR) in control and *E. alphitoides*-infected oak leaves collected at fixed time points (July, August and September) of three growing seasons. Values represent the means of 18 measurements per variant (C, <5% Inf, 12–15% Inf, 25% Inf) at each time point ±SD. Analysis of variance (ANOVA) for all parameters was followed by the Tukey post hoc test and statistically significant differences (*p* ≤ 0.05) are marked using low letters.

In the oak leaves infected with *E. alphitoides*, the highest increase in APX activity was observed in leaves collected in August (Figure 3). The results showed an approximately fourfold increase in APX activity in <5% Inf compared to the control. In July and September, increases in APX activity were observed in 12–15% Inf and <5% Inf, respectively, compared to the corresponding control.

The increase in DHAR activity was observed in <5% Inf leaves obtained in September compared to the control (Figure 3). In leaves obtained in July, a significant decrease in DHAR activity was observed in <5% Inf and 12–15% Inf compared to the control. In August, there were no significant changes in DHAR activity between control and infected leaves.

During the development of the infection, significant changes in MDHAR activity were observed in the leaves obtained from all time points (Figure 3). In July, the results showed an approximately twofold increase in MDHAR activity in <5% Inf and a lower increase in 12–15% Inf, as compared to the control. In August, a decrease in MDHAR activity was observed in 12–15% and 25% Inf compared to the control. In September, as a result of pathogen infection, significant increases in MDHAR activity were observed in <5% Inf, 12–15% Inf and 25% Inf compared to the control. The highest, approximately twofold increase in MDHAR activity was observed in 25% Inf.

In July, increased GR activity was observed in <5% Inf and 12–15% Inf compared to the control (Figure 3). In August, increased GR activity was observed in <5% and 12–15% Inf compared to the control. In the leaves obtained in September, significant increases in GR activity were observed in 12–15% and 25% Inf, respectively, compared to the control. The highest, an approximately threefold increase in GR activity, was observed in the 25% Inf group.

### 3.4. Infection-Related Changes in the Ascorbate and Glutathione Pools

The ascorbate content in oak leaves showed significant changes after pathogen infection (Table 2). In July, a significant increase in AsA content was observed in 12–15% of the infected leaves compared to the control. In other months, no significant AsA increases were observed in infected leaves compared to the respective controls. In leaves obtained in August, a significant increase in DHA content was observed in 12–15% Inf and 25% Inf compared to the control. In September, DHA content increased by <5% Inf and 25% Inf compared to the control. The highest AsA and DHA contents were observed in 25% Inf in August.

Pathogen infection caused significant changes in the glutathione pool at all time points (Table 3). In leaves obtained in July, a significant increase in GSH content was observed in 12–15% Inf compared to the control. In September, the results showed a significant increase in GSH and GSSG contents in 12–15% Inf and 25% Inf compared to the control. In July and August, GSSG levels were significantly higher than the control in <5% Inf and 12–15% Inf. The highest increase in GSSG content, approximately sevenfold, was observed in <5% Inf in July and tenfold in 12–15% Inf in August compared to the respective controls.

### 3.5. Changes in Lignin Content

**Figure 4:**
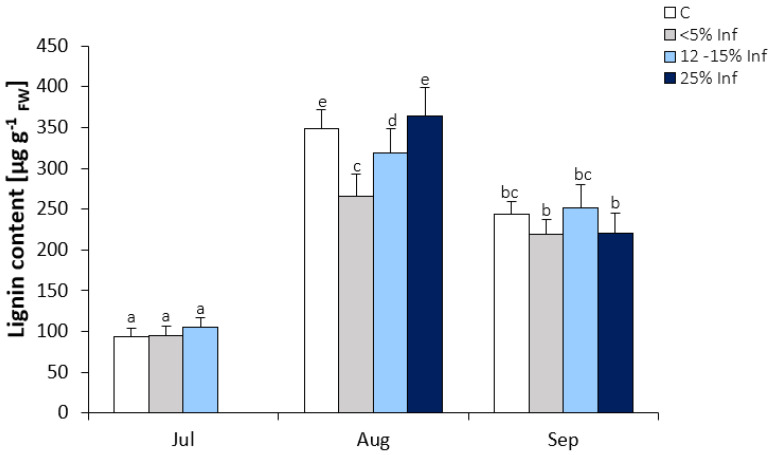
Lignin content in the control and *E. alphitoides* infected oak leaves collected at fixed time points (July, August, and September) of three growing seasons. Values represent the mean of 18 measurements per variant (C, <5% Inf, 12–15% Inf, 25% Inf) at each time point ±SD. Analysis of variance (ANOVA) for all parameters was followed by the Tukey post hoc test, and statistically significant differences (*p* ≤ 0.05) are marked using low letters.

A significant increase in lignin content was observed in leaves collected in August and September (Figure 4). In July, no significant changes in lignin content were observed in infected leaves compared to the control. In August, there was a significant decrease in the lignin content in <5% Inf and 12–15% Inf and no significant increase in 25% Inf compared to the control. In September, no significant changes in lignin content were observed between control and pathogen-infected leaves.

**Figure 5:**
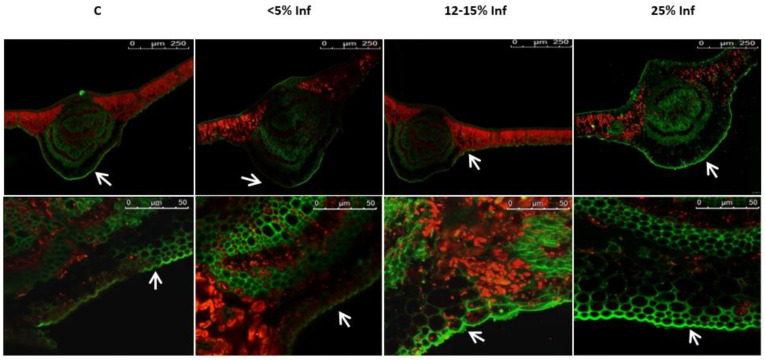
Detection and visualisation of lignin in control and *E. alphitoides*-infected oak leaves. A fluorescence confocal microscope (Leica TCS SP8; Leica Microsystems, Mannheim, Germany) was used to detect lignin deposition. White arrows indicate green fluorescence of lignin in the leaf epidermis analysed in August. Representative images were selected after analysing microscopic preparations of 3–4 plants from each experimental variant (C, <5% Inf, 12–15% Inf, 25% Inf).

Microscopic analysis revealed a fluorescence signal of lignin, which was located in the epidermis of both the control and the pathogen-infected leaves (Figure 5).

### 3.6. Changes in Different Phenolic Contents

As a result of *E. alphitoides* infection, significant changes in the content of phenolic compounds in oak leaves were observed (Table 4).

In July, a significant increase in total phenol content was observed in 12–15% Inf compared to the control. In leaves obtained in August, the decrease in total phenol content was observed in <5% Inf, 12–15% Inf, and 25% Inf. In September, a reduction in total phenol content was observed in 12–15% Inf and 25% Inf.

In the leaves obtained in July, an increase in the phenylpropanoid content was observed in 12–15% Inf compared to the control. In August and September, a decrease in phenylpropanoid content in 12–15% Inf and 25% Inf was observed compared to the control.

In July, an increase in flavonoid content was observed in 12–15% Inf compared to the control. In August, a decrease in flavonoid content was observed in <5% Inf, 12–15% Inf, and 25% Inf, compared to the control.

In the leaves obtained in August, an increase in anthocyanin content was observed in <5% Inf. At the other time points, no significant increase in anthocyanin content was observed compared to the respective controls.

## 4. Discussion

Trees may be subject to different patterns of pathogen pressure and require different types of protection. In our study, we show that the defensive responses of *Q. robur* leaves to biotic stress caused by powdery mildew are triggered during the three months of disease development, but the greatest changes are observed in August and September. Plants respond to biotrophs through a variety of morphological, biochemical and molecular mechanisms. These mechanisms, including components of the antioxidant system, elemental composition, and lignin content, are used to counter the effects of pathogen attack. [49].

Oszako et al. (2020) [50] investigated the production of secondary metabolites that may influence the interaction between *Trichoderma* species and the host *Q. robur*. They monitored physiological changes as a water index in oak seedlings showing symptoms of powdery mildew. The researchers showed that the antagonist fungus *T. asperellum* increased the total water content of oak leaves, which may serve as a preventive measure to reduce energy losses during water transpiration in infected plants, thus promising longer-term control of oak powdery mildew, especially in nurseries. Prosyannikova (2002) [51] found that infection of oak *Q. petraea* by the pathogenic fungus *E. alphitoides* is accompanied by pathological changes in the plant’s water regime, and the researcher also showed an increase in transpiration in diseased leaves. In our study, no significant changes in MC were observed in the infected leaves compared to the respective controls, indicating an undisturbed cellular water metabolism of *Q. robur* after *E. alphitoides* infection.

Defensive responses based on metal activity require the following conditions: the metal is more toxic to the pathogen or herbivore than to the plant; the metal inhibits the virulence of the pathogen or herbivore; and the metal increases the plant’s resistance to biotic stress [20,52]. The high content of elemental components seems to be able to substitute for defects in common biochemical defences or biotic stress signalling and protect plants [53]. Taking the above into account, metal accumulation within plant tissues can contribute to defence against biotic stress. Research on this subject indicates that the roles in plant defence are mainly documented for Mn, Ni, Cu, Fe, and Zn [54]. The results obtained in this study showed that the levels of Mn, Ni, Fe, Zn, and Mg were significantly increased in the infected oak leaves at different time points. Mn is involved in the production of phenolic compounds, the promotion of lignin and callose accumulation, and other plant defence mechanisms [55]. According to Eskandari et al. [56], Mn treatment against *Podosphaera fuliginea*, which causes powdery mildew, can induce biochemical changes, including increased peroxidase and polyphenol oxidase activities and the cell wall lignin content of cucumber leaves. In addition, the protective effect of Mn may be similar to that of Cu. For example, Mn reduces the susceptibility of plants to fungal or bacterial pathogens and is therefore often used to protect different crops against various infectious diseases [57]. Other metals may play different roles in plant function and the induction of defence responses. For example, Ni is involved in the plant antioxidant system during plant response to stress [58]; Fe contributes to pathogenesis-related mechanisms at the cellular level [59]; Mg is a component of the middle lamella and a component of the chlorophyll molecule [60]; and Ca^2+^ signalling plays both positive and negative roles in plant-pathogen interactions [61]. Zn plays an important role in plant growth, development, and defence against various stresses [62]. In addition, Zn is involved in plant-pathogen interactions, and its key role is the activation or stabilisation of metalloenzymes [63]. 

The defence responses against pathogens can include the oxidative burst and subsequent activation of cell death during HR. SOD catalyses the conversion of superoxide radicals to hydrogen peroxide, which is involved in biotic stress signalling. Cu/ZnSOD activity is generally increased in plants challenged by pathogens and is a dominant isoform in oak leaves [24]. In parallel to SOD, the ascorbate-glutathione cycle, which is active in all cellular compartments, plays a key role in the management of ROS generated under biotic stress and in redox signalling. In collaboration with SOD and CAT, the ascorbate-glutathione cycle strongly influences the interval-specific signature of ROS and, through redox-based communication with other signalling pathways, contributes to the complex regulatory mechanism of the plant stress response [64]. With regard to the ascorbate-glutathione cycle, the pathogen infection may cause changes in the activity of APX and reductases responsible for maintaining ascorbate and glutathione in a reduced form (GR, DHAR, and MDHAR) and may influence the content of the reduced and oxidised forms of ascorbate and glutathione, which may be important in the redox regulation of metabolism. The results of this study indicate an increase in the contents of ascorbate and glutathione in infected oak leaves, with some differences between metabolites, and therefore, they confirm stimulation of the Halliwell-Asada cycle under stress conditions. Similar results were obtained by Ghanbary et al. [65], who studied the effect of a combination of abiotic stress (drought stress) and biotic stress (*Biscogniauxia mediterranea* and *Obolarina perlica*) on biochemical changes in *Quercus brantii* Lindl seedlings. Ascorbate is oxidised by ROS and overgenerated in stress conditions, resulting in the formation of two oxidised forms of AsA, DHA, and MDHA. DHAR and MDHAR are enzymes involved in the regeneration of AsA [66,67]. This study showed an increase in the activity of the enzymes mentioned in leaves infected with the pathogen, mainly in seedlings obtained in September. Whereas, the increase in APX activity was demonstrated at the beginning of infection development. It can be assumed that in the cell, the regulation of the content of H_2_O_2,_ which may act as a signalling molecule of defence, depends on the activity of APX. However, in leaves with a large area of disease, changes in APX activity may be ineffective. In our previous research [10], it was found that the overproduction of H_2_O_2_ was accompanied by an increase in CAT activity, with a simultaneous decrease in APX activity, in the initial stage of infection, as well as an increase in SOD activity, in the later stage of infection development. The relationship was observed mainly in September, i.e., at the end of the experiment. GR is another enzyme, in addition to APX, involved in the Halliwell-Asada cycle, and its activity is essential to maintaining the levels of glutathione needed for dehydroascorbate reduction [67,68]. GR activity was much higher in leaves infected with the pathogen. The result obtained may be related to an increase in APX activity in infected leaves and may indicate an enhancement of the Halliwell-Asada cycle in seedlings infected with the pathogen.

Several studies have shown that the role of phenolic compounds in tree resistance is related to their involvement in oxidative processes in cells [69,70,71]. The influence of abiotic and biotic factors on the content of phenolic compounds and thus on plant resistance may be dependent on oxidative stress at the cellular level. Certain groups of phenolic compounds can act as antioxidants by donating electrons to peroxidases that are associated with, for example, lignification and defence against pathogens [72,73]. The generated phenolic radicals can then be reduced by ascorbate [74]. The aim of plant cells infected with the pathogen is to create effective mechanical barriers that prevent the pathogen from entering and at the same time deprive it of the possibility of further growth and development. The function of inhibiting the spread of the pathogen in the plant cell is performed by different phenolic compounds, with particular emphasis on lignin-forming components, belonging to the most persistent organic compounds [75,76]. Many reports indicate the participation of lignin or phenolic compounds involved in their formation in woody plants defence response to pathogens. For example, in *Eucalyptus nitens*, lignin deposition in the early stages of infection by *Mycosphaerella*, explains its greater resistance to the pathogen, compared to *Eucalyptus globulus* [77]. *Pinus nigra* infection by *Sphaeropsis sapinea* induces lignin deposition, which is associated with plant resistance to the pathogen [78]. Bucciarelli et al. [79] compared the histochemical responses in resistant stem tissues and susceptible genotypes of poplars (*Populus* sp.) inoculated with the fungus *Entoleuca mammata*, causing canker. In the resistant poplar genotype, infection was first limited by the formation of an intact, woody barrier around the site of infection and then by the development of a phenolic-rich callus at the wound site. In the case of the susceptible poplar genotype, no reactions stopping the infection were observed. These results suggest an important role of lignification in the defence strategy of poplars against *E. mammata*. Clark and Goldberg Oppenheimer (2024) [23] demonstrated the investigation of pathogen-host tissue interactions in trees, providing new insights into infections of *Q. robur* leaves by the fungus *E. alphitoides*. The researchers showed an increase in lignin peaks in both tissue types, indicating a defence-induced lignification of cell walls in response to the pathogen. This is most likely due to cell wall lignification, a plant defence response triggered by the presence of an invading pathogen, which is often a compensatory mechanism for the increased vulnerability of plant cells due to the loss of cellulose and pectin. In the present study, it was found that, along with the progressive infection in oak leaves, the lignin content does not increase. Lignin detection, by confocal fluorescence microscopy, showed the presence of lignin both in control leaves and those infected with the pathogen *E. alphitoides*. From the obtained data, the conclusion is that the host cells do not mobilise sufficiently lignin deposition to create an effective mechanical barrier that could contain the further spread of the pathogen. Despite the lack of significant differences in the deposition of lignin in control plants and those infected with *E. alphiotoides*, the previous studies using the HPLC technique [24] showed a significant increase in the content of precursors of lignin polymers, which might allow the strengthening of the mechanical barriers. The content of other phenols, i.e., phenylpropanoids, flavonoids, and anthocyanins, in leaves infected with the pathogen, showed slight changes compared to the control leaves, however, with a tendency to decrease their concentration after infection.

## 5. Conclusions

From all the points discussed above regarding the interaction between *Q. robur* and *E. alphitoides*, it can be concluded that (1) the activities of APX, DHAR, MDHAR, and GR; (2) the levels of ascorbate and glutathione, which are involved in promoting effective plant defence responses, were increased with increasing infection severity; and (3) elemental components such as Mn, Ni, Fe, Zn, and Mg, which accumulate to different degrees in infected leaves, may be key to the development of plant defence. The combination of our results shows that all detected compounds are associated with direct and indirect protection of *Quercus robur* leaves against *E. alphitoides*.

## Figures and Tables

**Table 1:** Properties of soil used for plant cultivation. The analyses were carried out in accordance with Polish standards and procedures.

Attribute	Value	Analytical Method	Analytical Procedure
pH in H_2_O	5.6	Potentiometric	PB 01 ed. 1 (14 May 2004)
Acidity in NaCl (g dm^3^ soil)	0.8	Conductometric	PB 02 ed. 2 (10 May 2016)
Available nutrients [mg dm^3^ soil]			
N-NO_3_	41.0	Potentiometric	PB 02 ed. 1 (14 May 2004)
N-NH_4_	21.0	Potentiometric	PB 02 ed. 1 (14 May 2004)
P (P_2_O_5_)	43.6	Spectrophotometric	PB 02 ed. 1 (14 May 2004)
K (K_2_O)	146	Atomic emission spectroscopy	PB 04 ed. 1 (21 May 2004)
Mg	492	Atomic absorption spectroscopy	PB 05 ed. 1 (28 May 2004)
Mn	3.5	Atomic absorption spectroscopy	PB 05 ed. 1 (28 May 2004)
Cu	2.6	Atomic absorption spectroscopy	PB 09 ed. 1 (5 May 2004)
Zn	4.6	Atomic absorption spectroscopy	PB 09 ed. 1 (5 May 2004)
Fe	41.4	Atomic absorption spectroscopy	PB 09 ed. 1 (5 May 2004)
Ca	679	Atomic emission spectroscopy	PB 04 ed. 1 (21 May 2004)

**Table 2:** Effect of *E. alphitoides* infection on the ascorbate pool in oak leaves collected at fixed times (July, August, and September) of the three growing seasons. Values represent the mean of 18 measurements per variant (C, <5% Inf, 12–15% Inf, 25% Inf) at each time point ±SD. Analysis of variance (ANOVA) for all parameters was followed by the Tukey post hoc test, and statistically significant differences (*p* ≤ 0.05) are marked using low letters.

Ascorbate Pool	Time	Plant Variants			
[μmol g^−1^_FW_]	Month	C	<5% Inf	12–15% Inf	25% Inf
AsA + DHA	July	26.1 ± 1.2 a	27.4 ± 1.0 a	31.6 ± 1.0 bc	˗
	August	44.7 ± 3.1 e	34.3 ± 2.1 c	47.7 ± 1.8 f	59.3 ± 2.3 g
	September	31.3 ± 2.0 b	39.3 ± 1.5 d	28.3 ± 1.0 a	33.5 ± 2.9 bc
AsA	July	20.6 ± 3.6 ab	20.7 ± 1.6 ab	25.72 ± 3.2 c	˗
	August	32.5 ± 1.0 e	25.4 ± 2.5 c	28.97 ± 2.2 d	32.8 ± 2.1 e
	September	21.1 ± 2.5 ab	22.9 ± 3.1 bc	19.58 ± 1.22 a	20.2 ± 1.7 ab
DHA	July	5.5 ± 0.7 a	6.7 ± 1.2 ab	5.9 ± 1.9 ab	˗
	August	12.2 ± 1.0 de	8.8 ± 2.1 bc	18.7 ± 1.2 f	26.5 ± 1.6 g
	September	10.2 ± 1.5 cd	16.4 ± 1.2 f	8.7 ± 0.4 bc	13.4 ± 0.8 e

AsA—ascorbate, reduced form, DHA—dehydroascorbate, oxidised form.

**Table 3:** Effect of *E. alphitoides* infection on the glutathione pool in oak leaves collected at fixed times (July, August, and September) of three growing seasons. Values represent the mean of 18 measurements per variant (C, <5% Inf, 12–15% Inf, 25% Inf) at each time point ±SD. Analysis of variance (ANOVA) for all parameters was followed by the Tukey post hoc test, and statistically significant differences (*p* ≤ 0.05) are marked using low letters.

Glutathione Pool	Time	Plant Variants			
[μmol g^−1^_FW_]	Month	C	<5% Inf	12–15% Inf	25% Inf
GSH + GSSG	July	30.5 ± 0.5 c	40.7 ± 2.8 d	51.1 ± 0.6 e	˗
	August	33.1 ± 0.7 c	30.0 ± 0.8 bc	61.4 ± 0.6 f	21.2 ± 0.6 a
	September	26.8 ± 2.8 abc	23.7 ± 1.2 ab	70.1 ± 5.9 g	52.0 ± 1.3 e
GSH	July	28.8 ± 0.4 cd	33.3 ± 2.8 def	47.8 ± 0.6 g	˗
	August	30.8 ± 0.7 cde	26.2 ± 0.7 bc	36.1 ± 0.5 ef	20.5 ± 0.5 ab
	September	17.9 ± 2.9 a	19.9 ± 1.3 ab	54.5 ± 5.9 h	37.4 ± 1.4 f
GSSG	July	1.6 ± 0.1 b	7.4 ± 0.1 f	3.3 ± 0.1 d	˗
	August	2.3 ± 0.1 c	3.9 ± 0.1 e	25.3 ± 0.1 j	0.7 ± 0.1 a
	September	8.9 ± 0.1 g	3.8 ± 0.1 e	15.7 ± 0.1 i	14.5 ± 0.1 h

GSH—glutathione, reduced form; GSSG—glutathione disulphide, oxidised form.

**Table 4:** Effect of *E. alphitoides* infection on different phenolic compounds in oak leaves collected at fixed time points (July, August, and September) of three growing seasons. Values represent the mean of 18 measurements per variant (C, <5% Inf, 12–15% Inf, 25% Inf) at each time point ±SD. Analysis of variance (ANOVA) for all parameters was followed by the Tukey post hoc test, and statistically significant differences (*p* ≤ 0.05) are marked using low letters.

Phenolic Compounds	Time	Plant Variants			
	Month	C	<5% Inf	12–15% Inf	25% Inf
Total phenols	July	18.5 ± 1.0 a	19.7 ± 1.2 ab	22.8 ± 1.2 bc	˗
[mg g^−1^_FW_]	August	30.4 ± 1.0 e	24.6 ± 1.1 cd	18.5 ± 1.1 a	24.6 ± 1.1 cd
	September	24.9 ± 1.3 d	24.6 ± 1.4 cd	17.6 ± 1.1 a	22.7 ± 1.2 bc
Phenylpropanoids	July	16.4 ± 1.0 abc	14.7 ± 1.2 a	20.9 ± 1.4 e	˗
[mg g^−1^_FW_]	August	21.5 ± 1.1 de	20.5 ± 0.7 de	16.5 ± 1.4 abc	17.5 ± 1.7 abc
	September	19.0 ± 0.7 cde	17.9 ± 1.4 bcd	15.0 ± 0.8 ab	16.1 ± 0.8 ab
Flavonoids	July	160 ± 4 a	140 ± 7 a	330 ± 18 b	˗
[μg g^−1^_FW_]	August	330 ± 74 b	180 ± 10 a	190 ± 10 a	200 ± 11 a
	September	180 ± 12 a	170 ± 10 a	110 ± 13 a	130 ± 10 a
Anthocyanin	July	5.9 ± 1.3 ab	7.4 ± 1.0 bc	4.5 ± 1.0 a	˗
[μg g^−1^_FW_]	August	5.6 ± 1.1 ab	5.2 ± 0.9 ab	7.9 ± 1.3 bc	7.9 ± 1.3 bc
	September	5.5 ± 1.1 ab	8.9 ± 1.3 c	5.8 ± 1.3 ab	7.1 ± 0.8 abc

## Data Availability

The original contributions presented in the study are included in the article, further inquiries can be directed to the corresponding author.
